# Association between walking ability and trunk and lower-limb muscle atrophy in institutionalized elderly women: a longitudinal pilot study

**DOI:** 10.1186/s40101-015-0069-z

**Published:** 2015-08-28

**Authors:** Tome Ikezoe, Masatoshi Nakamura, Hiroto Shima, Yasuyoshi Asakawa, Noriaki Ichihashi

**Affiliations:** Human Health Sciences, Graduate School of Medicine, Kyoto University, 53 Shogoin Kawahara-cho, Sakyo-ku, Kyoto, 606-8507 Japan; Faculty of Health and Sports Sciences, Doshisha University, Kyoto, Japan; Takeda Hospital, Kyoto, Japan; Graduate School of Human Health Sciences, Tokyo Metropolitan University, Tokyo, Japan

**Keywords:** Aging, Muscle atrophy, Ultrasonography, Elderly women, Walking ability

## Abstract

**Background:**

The aim of this study was to investigate the association between walking ability and muscle atrophy in the trunk and lower limbs.

**Methods:**

Subjects in this longitudinal study were 21 elderly women who resided in nursing homes. The thicknesses of the following trunk and lower-limb muscles were measured using B-mode ultrasound: rectus abdominis, external oblique, internal oblique, transversus abdominis, erector spinae, lumbar multifidus, psoas major, gluteus maximus, gluteus medius, gluteus minimus, rectus femoris, vastus lateralis, vastus intermedius, biceps femoris, gastrocnemius, soleus, and tibialis anterior. Maximum walking speed was used to represent walking ability. Maximum walking speed and muscle thickness were assessed before and after a 12-month period.

**Results:**

Of the 17 measured muscles of the trunk and lower limbs, age-related muscle atrophy in elderly women was greatest in the erector spinae, rectus femoris, vastus lateralis, vastus intermedius, and tibialis anterior muscles. Correlation coefficient analyses showed that only the rate of thinning of the vastus lateralis was significantly associated with the rate of decline in maximum walking speed (*r* = 0.518, *p* < 0.05).

**Conclusions:**

This longitudinal study suggests that reduced walking ability may be associated with muscle atrophy in the trunk and lower limbs, especially in the vastus lateralis muscle, among frail elderly women.

## Introduction

There are numerous studies on age-related muscle atrophy using ultrasonographic measurements of muscle thickness. Strong correlations have been reported between muscle thickness measured by B-mode ultrasound and site-matched skeletal muscle mass measured by magnetic resonance imaging [1–4]. Therefore, measurements of muscle thickness using ultrasound can be used to noninvasively estimate the degree of muscle atrophy [5]. Kubo et al. [6] reported that the thicknesses of the vastus lateralis and medial gastrocnemius muscles, as measured by B-mode ultrasound, decreased significantly with advancing age, whereas no significant age-related change in muscle thickness was observed for the triceps brachii muscle. Our previous study using ultrasound revealed relatively little atrophy of the soleus but marked atrophy of the psoas major among lower-limb muscles in elderly women able to walk without assistance [7].

With regard to age-related changes in the size of trunk muscles, Rankin et al. [8] reported a significant negative correlation between age and thickness of the abdominal muscles in healthy subjects aged 20–72 years. Kanehisa et al. [9] also found that the rectus abdominis muscle was significantly thinner in elderly than in younger patients. Ota et al. [10] demonstrated that age-related muscle atrophy of the rectus abdominis begins at an early age and that less age-related atrophy is seen in the deep abdominal muscles such as the transversus abdominis. We also reported less age-related atrophy in the deep trunk muscles (transversus abdominis and lumbar multifidus) of elderly women who were able to walk [11]. Thus, although age-related changes in individual muscles have been reported in cross-sectional studies, age-related changes in trunk and lower-limb muscles have not been examined in longitudinal studies.

Age-related muscle atrophy (sarcopenia) is a key component of the frailty syndrome observed in the elderly [12], while the prevalence of sarcopenia is high in long-term care populations compared with community-dwelling populations [13]. In frail elderly people who are institutionalized, decreases in motor function from a lack of specialized exercise training may occur in as little as 12 months and may lead to difficulty ambulating. In fact, there is evidence of a relationship between locomotor ability and thinning of the muscles of the trunk and lower extremities among the frail elderly. Our previous studies demonstrated that the degree of daily physical activity was associated with the thickness of the gluteus medius muscle in frail elderly women [7] and that marked muscle atrophy was seen in the quadriceps femoris muscles of elderly women who did not walk for a long period [14]. Further, we reported that in elderly women who were not able to ambulate independently or perform other activities of daily living, atrophy of the trunk muscles was more marked among the antigravity muscles, such as the back muscles and transversus abdominis [11]. In these cross-sectional studies, it remains unclear whether muscle atrophy led to reduced walking ability or whether muscle atrophy progressed as a result of inactivity resulting from reduced walking ability. Therefore, assessments of the changes in muscle mass and walking ability are required to clarify causality. However, there are no longitudinal studies focusing on the influence of walking ability among elderly people on trunk and lower-limb muscle atrophy. Thus, the aim of this study was to investigate age-related changes in individual muscle thickness and the relationship between walking ability and the decline in muscle thickness of the trunk and lower extremities over time among institutionalized elderly women.

## Methods

### Subjects

Subjects were 21 elderly women (mean age 82.4 ± 6.5 years). All subjects were residents of nursing homes in Kyoto, Japan, who were able to ambulate independently or with an assistive device and were without unstable physical conditions. Patients with physical dysfunctions that may have influenced outcome measures, such as acute neurological impairment (e.g., acute stroke, Parkinson’s disease, and paresis of the lower limbs) or severe musculoskeletal or cognitive impairment, were excluded.

Our previous study examining habitual physical activity during the daytime in elderly women who resided in nursing homes such as those in the present study showed that institutionalized elderly subjects were taking <2000 steps/day and that they spent most of the day in sedentary, inactive positions such as sitting and lying [15]. Therefore, at least in this population, institutionalized elderly women have low habitual physical activity levels and “frailty.”

Subjects were informed about the study procedures before testing and provided their written informed consent before participating. The study was performed in accordance with the Declaration of Helsinki and was approved by Kyoto University Graduate School and the faculty of the medical ethics committee.

### Measurements of muscle thickness

Muscle thickness was measured using B-mode ultrasound imaging (LOGIQ Book XP; GE Healthcare Japan, Tokyo, Japan) with transducers with a range of 5–10 MHz. Seven trunk muscles were examined on the right side: the rectus abdominis, external oblique, internal oblique, transversus abdominis, erector spinae, lumbar multifidus, and psoas major. The measurement site of each muscle and patient position during measurement are shown in Table 1. Previous studies have demonstrated the high reliability of the ultrasound technique in measuring the thickness of trunk muscles [11, 16–19]. As abdominal muscle thickness increases during expiration [20–22], recordings were made at a consistent point at the end of relaxed expiration (when the respiratory muscles are relaxed) during the measurements of abdominal muscle thickness.

**Table 1:** Measurement positions and measurement sites for trunk muscles

Muscles	Positions	Measurement sites
Rectus abdominis	Supine	3 cm lateral to the umbilicus
External oblique	Supine	2.5 cm anterior to the mid-axillary line, at the midpoint between the inferior rib and the iliac crest
Internal oblique	Supine	2.5 cm anterior to the mid-axillary line, at the midpoint between the inferior rib and the iliac crest
Transversus abdominis	Supine	2.5 cm anterior to the mid-axillary line, at the midpoint between the inferior rib and the iliac crest
Erector spinae	Prone	7 cm lateral to the L3 spinous process
Lumbar multifidus	Prone	2 cm lateral to the L4 spinous process
Psoas major	Prone	7 cm lateral from the L3 spinous process

Ten lower-limb muscles were examined on the right side: the gluteus maximus, gluteus medius, gluteus minimus, rectus femoris, vastus lateralis, vastus intermedius, biceps femoris, gastrocnemius, soleus, and tibialis anterior. The measurement site of each muscle and patient position during measurement are shown in Table 2. Previous studies have shown high reliability of the ultrasound technique in the measurement of lower-limb muscle thickness [14, 23–26].

**Table 2:** Positions and sites of measurement of lower-limb muscles

Muscles	Positions	Measurement sites
Gluteus maximus	Prone	30 % proximal between the posterior superior iliac spine and the greater trochanter
Gluteus medius	Prone	Midway between the proximal end of the iliac crest and the greater trochanter
Gluteus minimus	Prone	Midway between the proximal end of the iliac crest and the greater trochanter
Rectus femoris	Supine	Midway between the anterior superior iliac spine and the proximal end of the patella
Vastus lateralis	Supine	3 cm lateral of 60 % distal between the anterior superior iliac spine and the proximal end of the patella
Vastus intermedius	Supine	Midway between the anterior superior iliac spine and the proximal end of the patella
Biceps femoris	Prone	Midway between the ischial tuberosity and the lateral condyle of the tibia
Gastrocnemius	Prone	Medial head of gastrocnemius at 30 % proximal between the lateral malleolus of the fibula and the lateral condyle of the tibia
Soleus	Prone	30 % proximal between the lateral malleolus of the fibula and the lateral condyle of the tibia
Tibialis anterior	Supine	30 % proximal between the lateral malleolus of the fibula and the lateral condyle of the tibia

During the examination, care was taken to maintain the same standardized position of the subjects and the exact location of the transducer. To improve acoustic coupling, a water-soluble transmission gel was placed over the scan head. The transducer was held perpendicular to the skin surface using the minimum pressure required to achieve a clear image. All muscle thicknesses were assessed once at the beginning of the study and once 12 months later.

### Assessment of walking ability

Maximum walking speed, which was used to represent walking ability, was measured over a distance of 5 m. Participants were provided an additional 2 m to accelerate before the test distance and 2 m to decelerate afterwards and were asked to walk as fast as they could. Two trials were performed, and the faster speed (m/s) was used for the analyses. Maximum walking speed was assessed once at the beginning of the study and once 12 months later.

### Statistical analysis

All data are presented as mean ± standard deviation (SD). Differences in maximum walking speed and muscle thickness between baseline and 12 months later were examined using a Wilcoxon signed-rank test. We calculated the percent change in maximum walking speed and percent change in muscle thickness using the following formula: percent change = (value after 12 months − baseline value) × (baseline value)^−1^ × 100. Spearman’s rank correlation coefficient was used to investigate the relationship between rates of change in maximum walking speed and muscle thickness. Significance was set at *p* < 0.05.

## Results

### Changes in maximum walking speed and muscle thickness between baseline and 12 months later

No major health problems, including acute neurological or severe musculoskeletal complications or significant weight loss, occurred during the follow-up period. The maximum walking speed at 12 months later was significantly slower than that at baseline (−12.7 ± 14.9 %, *p* < 0.01) (Table 3).

**Table 3:** Changes in characteristics and maximum walking speed between baseline and 12 months later

	Baseline	12 months later
Height (m)	1.48 ± 0.08	1.48 ± 0.09
Weight (kg)	47.0 ± 8.7	46.1 ± 8.7
Body mass index (kg/m^2^)	21.4 ± 3.4	21.1 ± 3.4
Maximum walking speed (m/s)	1.28 ± 0.37	1.10 ± 0.32**

**p < 0.01 compared with baseline

Table 4 shows muscle thicknesses (mean ± SD) at baseline and 12 months later. The thicknesses of the erector spinae, rectus femoris, vastus lateralis, vastus intermedius, and tibialis anterior muscles decreased significantly over the 12-month period. There were no significant differences in the thicknesses of the rectus abdominis, external oblique, internal oblique, transversus abdominis, lumbar multifidus, psoas major, gluteus maximus, gluteus medius, gluteus minimus, biceps femoris, gastrocnemius, and soleus muscles.

**Table 4:** Changes in muscle thicknesses between baseline and 12 months later

Muscles	Baseline (mm)	12 months later (mm)	Percent change (%)
Rectus abdominis	6.2 ± 1.9	6.1 ± 1.9	0.4 ± 16.4
External oblique	4.9 ± 1.2	4.9 ± 1.5	1.6 ± 22.5
Internal oblique	7.1 ± 2.0	6.8 ± 2.5	−1.1 ± 33.1
Transversus abdominis	3.2 ± 1.0	3.1 ± 1.1	0.8 ± 23.3
Erector spinae	26.6 ± 6.4	21.9 ± 5.7*	−12.0 ± 35.6
Lumbar multifidus	26.8 ± 5.8	26.3 ± 6.3	1.1 ± 25.6
Psoas major	13.7 ± 5.2	11.6 ± 3.1	−5.4 ± 35.7
Gluteus maximus	15.3 ± 4.0	14.8 ± 4.8	−1.7 ± 23.4
Gluteus medius	15.5 ± 4.4	15.2 ± 5.0	−0.1 ± 27.2
Gluteus minimus	12.3 ± 3.9	12.3 ± 4.0	1.6 ± 30.2
Rectus femoris	16.5 ± 4.2	11.5 ± 4.2**	−28.3 ± 25.0
Vastus lateralis	13.2 ± 3.9	10.6 ± 3.4**	−17.3 ± 25.2
Vastus intermedius	10.8 ± 3.1	8.4 ± 2.8**	−19.8 ± 22.9
Biceps femoris	18.3 ± 4.6	17.3 ± 5.5	−2.6 ± 30.8
Gastrocnemius	11.3 ± 3.3	10.9 ± 2.4	1.8 ± 26.4
Soleus	29.3 ± 6.8	28.3 ± 5.5	−0.6 ± 20.8
Tibialis anterior	21.8 ± 3.1	18.7 ± 2.5**	−12.8 ± 14.0

*p < 0.05 compared with baseline; **p < 0.01 compared with baseline

### Percent change in muscle thickness compared with baseline

Table 4 shows the percent change in muscle thickness for each muscle between baseline and 12 months later. Thinning was greatest for the rectus femoris (−28.3 ± 25.0 %), followed by the vastus intermedius (−19.8 ± 22.9 %), vastus lateralis (−17.3 ± 25.2 %), tibialis anterior (−12.8 ± 14.0 %), and erector spinae (−12.0 ± 35.6 %).

### Relationship between change in maximum walking speed and change in muscle thickness

**Fig. 1:**
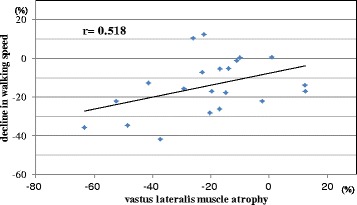
Relationship between percent change in walking speed and vastus lateralis muscle atrophy

Table 5 shows the relationship between percent change in maximum walking speed and percent change in the thickness of each muscle. Spearman’s rank correlation coefficient analyses showed that only the percent change in vastus lateralis muscle thickness was significantly associated with the percent change in maximum walking speed (*r* = 0.518, *p* < 0.05) (Fig. 1).

**Table 5:** Relationships between percent change in maximum walking speed and percent change in muscle thickness

	Correlation coefficients	*p* value
Rectus abdominis	0.041	0.859
External oblique	0.409	0.065
Internal oblique	0.415	0.061
Transversus abdominis	0.337	0.135
Erector spinae	0.412	0.064
Lumbar multifidus	0.094	0.685
Psoas major	0.195	0.410
Gluteus maximus	0.098	0.709
Gluteus medius	−0.025	0.924
Gluteus minimus	0.128	0.625
Rectus femoris	0.399	0.073
Vastus lateralis	0.518	0.016*
Vastus intermedius	0.360	0.109
Biceps femoris	0.203	0.390
Gastrocnemius	0.086	0.719
Soleus	0.247	0.293
Tibialis anterior	0.008	0.974

*Significant correlation at *p* < 0.05

## Discussion

There were two main findings in this longitudinal study. First, of the 17 muscles of the trunk and lower limbs measured, age-related muscle atrophy in frail elderly women was greatest for the erector spinae, tibialis anterior, and quadriceps femoris muscles such as the rectus femoris, vastus lateralis, and vastus intermedius muscles. Second, walking ability was associated with age-related muscle atrophy in the vastus lateralis muscle among frail elderly women. To our knowledge, this is the first longitudinal report to demonstrate age-related declines in individual muscles of the trunk and lower limbs and the relationship between walking ability and sarcopenia in the trunk and lower-limb muscles.

The muscles of the trunk and lower limbs play important roles in stabilizing the body, maintaining posture, and controlling spinal and lower-limb movement. Therefore, prevention of age-related atrophy of these muscles is important for maintaining the ability of the elderly to perform activities of daily living. In this study, measurement of the thicknesses of 17 muscles of the trunk and lower limbs revealed no significant differences between baseline and 12 months later in the thicknesses of the deep muscles, such as the transversus abdominis, lumbar multifidus, and soleus. These findings are consistent with those from our previous cross-sectional studies [11, 14]. In general, only moderate age-related losses occur in the deep muscles, which are made up of a larger proportion of type I fibers [27–29]. The deep muscles of the trunk and lower limbs can be maintained by a small amount of muscle contraction during daily physical activities, without any special exercise training and regardless of the aging process, in elderly people who are able to walk independently. Our results suggest, however, that age-related atrophy may occur in the erector spinae, quadriceps femoris, and tibialis anterior muscle in as little as 12 months.

In our previous cross-sectional study of elderly subjects who had not walked for a long period of time, we found that muscle atrophy was greatest in the quadriceps muscles [14]. However, it remains unclear whether muscle atrophy of the quadriceps femoris progressed as a result of inactivity or whether muscle atrophy of quadriceps femoris led to disability. The results of the present longitudinal study also showed that a decline in walking ability was associated with progression of muscle atrophy, especially in the vastus lateralis, in the trunk and lower-limb muscles. Reduced physical activity leads to a loss of skeletal muscle mass. Lower-limb muscles, especially antigravity muscles such as the quadriceps femoris, which are required for weight-bearing activities (i.e., walking and stair climbing), are likely to be most affected by inactivity and reduced gravitational loading. Previous studies have documented that muscle mass in the human quadriceps femoris was reduced by 3 % after 7 days [30], 8 % after 20 days [31], and 14 % after 42 days [32] of bed rest. Thus, loss of muscle mass in the quadriceps femoris is exacerbated by periods of inactivity.

Our results indicate that of the quadriceps femoris muscle group, only the vastus lateralis was associated with walking speed. The vastus lateralis muscle is important in controlling knee joint angle during gait. The physiological cross-sectional area and knee moment arm of the vastus lateralis muscle are larger than those of the vastus intermedius and rectus femoris muscles [33, 34]. Our results suggest that reduced walking ability associated with muscle atrophy, especially in the vastus lateralis muscle, may lead to further declines in walking ability.

This study has several limitations. The first relates to our measurement of muscle thickness for determining muscle mass. In general, muscle cross-sectional area is thought to reflect muscle size more accurately. However, the obtained measurement values did not reflect both the longitudinal axis and transverse axis as we took measurements in only one dimension. Another limitation of this longitudinal study is its small subject sample size and a short duration of 12 months. Further, we were unable to determine the actual cause of the relationship between muscle atrophy and walking ability, as we did not examine the differences between more active and less active subjects. Future longitudinal studies with larger sample sizes and of longer duration are required to further clarify the association between the degree of decline in muscle mass and walking ability, including lifestyle factors such as habitual physical activity and sedentary behavior outcomes, in the elderly.

In conclusion, this longitudinal study suggests that age-related muscle atrophy was greatest in the erector spinae, quadriceps femoris, and tibialis anterior muscles among trunk and lower-limb muscles. Additionally, our findings suggest that reduced walking ability exacerbates age-related muscle atrophy in the trunk and lower limbs, especially in the vastus lateralis muscle, among frail elderly women.

## References

[1] Dupont AC, Sauerbrei EE, Fenton PV, Shragge PC, Loeb GE, Richmond FJ (2001). Real-time sonography to estimate muscle thickness: comparison with MRI and CT. J Clin Ultrasound.

[2] Fukunaga T, Miyatani M, Tachi M, Kouzaki M, Kawakami Y, Kanehisa H (2001). Muscle volume is a major determinant of joint torque in humans. Acta Physiol Scand.

[3] Miyatani M, Kanehisa H, Ito M, Kawakami Y, Fukunaga T (2004). The accuracy of volume estimates using ultrasound muscle thickness measurements in different muscle groups. Eur J Appl Physiol.

[4] Sanada K, Kearns C, Midorikawa T, Abe T (2006). Prediction and validation of total and regional skeletal muscle mass by ultrasound in Japanese adults. Eur J Appl Physiol.

[5] Heymsfield SB, Gonzalez MC, Lu J, Jia G, Zheng J (2015). Skeletal muscle mass and quality: evolution of modern measurement concepts in the context of sarcopenia. Proc Nutr Soc.

[6] Kubo K, Kanehisa H, Azuma K, Ishizu M, Kuno SY, Okada M, Fukunaga T (2003). Muscle architectural characteristics in women aged 20–79 years. Med Sci Sports Exerc.

[7] Ikezoe T, Mori N, Nakamura M, Ichihashi N (2011). Age-related muscle atrophy in the lower extremities and daily physical activity in elderly women. Arch Gerontol Geriatr.

[8] Rankin G, Stokes M, Newham DJ (2006). Abdominal muscle size and symmetry in normal subjects. Muscle Nerve.

[9] Kanehisa H, Miyatani M, Azuma K, Kuno S, Fukunaga T (2004). Influences of age and sex on abdominal muscle and subcutaneous fat thickness. Eur J Appl Physiol.

[10] Ota M, Ikezoe T, Kaneoka K, Ichihashi N (2012). Age-related changes in the thickness of the deep and superficial abdominal muscles in women. Arch Gerontol Geriatr.

[11] Ikezoe T, Mori N, Nakamura M, Ichihashi N (2012). Effects of age and inactivity due to prolonged bed rest on atrophy of trunk muscles. Eur J Appl Physiol.

[12] Milte R, Crotty M (2014). Musculoskeletal health, frailty and functional decline. Best Pract Res Clin Rheumatol.

[13] Cruz Jentoft AJ, Landi F, Schneider SM, Zúñiga C, Arai H, Boirie Y, Chen LK, Fielding RA, Martin FC, Michel JP, Sieber C, Stout JR, Studenski SA, Vellas B, Woo J, Zamboni M, Cederholm T (2014). Prevalence of and interventions for sarcopenia in ageing adults: a systematic review. Report of the International Sarcopenia Initiative (EWGSOP and IWGS). Age Ageing.

[14] Ikezoe T, Mori N, Nakamura M, Ichihashi N (2011). Atrophy of the lower limbs in elderly women: is it related to walking ability?. Eur J Appl Physiol.

[15] Ikezoe T, Asakawa Y, Shima H, Kishibuchi K, Ichihashi N (2013). Daytime physical activity patterns and physical fitness in institutionalized elderly women: an exploratory study. Arch Gerontol Geriatr.

[16] Hebert JJ, Koppenhaver SL, Parent EC, Fritz JM (2009). A systematic review of the reliability of rehabilitative ultrasound imaging for the quantitative assessment of the abdominal and lumbar trunk muscles. Spine.

[17] Koppenhaver SL, Hebert JJ, Fritz JM, Parent EC, Teyhen DS, Magel JS (2009). Reliability of rehabilitative ultrasound imaging of the transversus abdominis and lumbar multifidus muscles. Arch Phys Med Rehabil.

[18] Stokes M, Hides J, Elliott J, Kiesel K, Hodges P (2007). Rehabilitative ultrasound imaging of the posterior paraspinal muscles. J Orthop Sports Phys Ther.

[19] Wallwork TL, Hides JA, Stanton WR (2007). Intrarater and interrater reliability of assessment of lumbar multifidus muscle thickness using rehabilitative ultrasound imaging. J Orthop Sports Phys Ther.

[20] Ainscough-Potts AM, Morrissey MC, Critchley D (2006). The response of the transverse abdominis and internal oblique muscles to different postures. Man Ther.

[21] De Troyer A, Estenne M, Ninane V, Van Gansbeke D, Gorini M (1990). Transversus abdominis muscle function in humans. J Appl Physiol.

[22] Misuri G, Colagrande S, Gorini M, Iandelli I, Mancini M, Duranti R, Scano G (1997). In vivo ultrasound assessment of respiratory function of abdominal muscles in normal subjects. Eur Respir J.

[23] Blazevich AJ, Gill ND, Zhou S (2006). Intra- and intermuscular variation in human quadriceps femoris architecture assessed in vivo. J Anat.

[24] Kellis E, Galanis N, Natsis K, Kapetanos G (2009). Validity of architectural properties of the hamstring muscles: correlation of ultrasound findings with cadaveric dissection. J Biomech.

[25] Thoirs K, English C (2009). Ultrasound measures of muscle thickness: intra-examiner reliability and influence of body position. Clin Physiol Funct Imaging.

[26] Maganaris CN, Baltzopoulos V, Sargeant AJ (1998). In vivo measurements of the triceps surae complex architecture in man: implications for muscle function. J Physiol.

[27] Doherty TJ, Vandervoort AA, Brown WF (1993). Effects of ageing on the motor unit: a brief review. Can J Appl Physiol.

[28] Lexell J, Taylor CC, Sjostrom M (1988). What is the cause of the ageing atrophy? Total number, size and proportion of different fiber types studied in whole vastus lateralis muscle from 15- to 83-year-old men. J Neurol Sci.

[29] Roos MR, Rice CL, Vandervoort AA (1997). Age-related changes in motor unit function. Muscle Nerve.

[30] Ferrando AA, Stuart CA, Brunder DG, Hillman GR (1995). Magnetic resonance imaging quantitation of changes in muscle volume during 7 days of strict bed rest. Aviat Space Environ Med.

[31] Akima H, Kuno S, Suzuki Y, Gunji A, Fukunaga T (1997). Effects of 20 days of bed rest on physiological cross-sectional area of human thigh and leg muscles evaluated by magnetic resonance imaging. J Gravit Physiol.

[32] Berg HE, Larsson L, Tesch PA (1997). Lower limb skeletal muscle function after 6 wk of bed rest. J Appl Physiol.

[33] Erskine RM, Jones DA, Maganaris CN, Degens H (2009). In vivo specific tension of the human quadriceps femoris muscle. Eur J Appl Physiol.

[34] Wilson NA, Sheehan FT (2009). Dynamic in vivo 3-dimensional moment arms of the individual quadriceps components. J Biomech.

